# Cellular and Molecular Changes Associated with Onion Skin Formation Suggest Involvement of Programmed Cell Death

**DOI:** 10.3389/fpls.2016.02031

**Published:** 2017-01-09

**Authors:** Ortal Galsurker, Adi Doron-Faigenboim, Paula Teper-Bamnolker, Avinoam Daus, Yael Fridman, Amnon Lers, Dani Eshel

**Affiliations:** ^1^Department of Postharvest Science of Fresh Produce, The Volcani Center, Agricultural Research OrganizationRishon LeZion, Israel; ^2^The Robert H. Smith Institute of Field Crops and Vegetables, Robert H. Smith Faculty of Agriculture Food and Environment, The Hebrew University of JerusalemRehovot, Israel; ^3^Institute of Plant Sciences, The Volcani Center, Agricultural Research OrganizationRishon LeZion, Israel; ^4^The Alexander Silberman Institute of Life Science, Edmond Safra Campus (G Ram), The Hebrew UniversityJerusalem, Israel

**Keywords:** DNA fragmentation, onion-skin, programmed cell death, scale, transcriptome

## Abstract

Skin formation of onion (*Allium cepa* L.) bulb involves scale desiccation accompanied by scale senescence, resulting in cell death and tissue browning. Understanding the mechanism of skin formation is essential to improving onion skin and bulb qualities. Although onion skin plays a crucial role in postharvest onion storage and shelf life, its formation is poorly understood. We investigated the mode of cell death in the outermost scales that are destined to form the onion skin. Surprisingly, fluorescein diacetate staining and scanning electron microscopy indicated that the outer scale desiccates from the inside out. This striking observation suggests that cell death in the outer scales, during skin formation, is an internal and organized process that does not derive only from air desiccation. DNA fragmentation, a known hallmark of programmed cell death (PCD), was revealed in the outer scales and gradually decreased toward the inner scales of the bulb. Transmission electron microscopy further revealed PCD-related structural alterations in the outer scales which were absent from the inner scales. *De novo* transcriptome assembly for three different scales: 1st (outer), 5th (intermediate) and 8th (inner) fleshy scales identified 2,542 differentially expressed genes among them. GO enrichment for cluster analysis revealed increasing metabolic processes in the outer senescent scale related to defense response, PCD processes, carbohydrate metabolism and flavonoid biosynthesis, whereas increased metabolism and developmental growth processes were identified in the inner scales. High expression levels of PCD-related genes were identified in the outer scale compared to the inner ones, highlighting the involvement of PCD in outer-skin development. These findings suggest that a program to form the dry protective skin exists and functions only in the outer scales of onion.

## Introduction

The onion (*Allium cepa* L.) bulb is considered to be one of the most important vegetable crops in the world (Hoffman, 1933; Griffiths et al., 2002; Mallor et al., 2011). The onion plant’s leaves grow in a circular pattern from a flattened stem, giving rise to older leaves on the outside and younger leaves on the inside (Hoffman, 1933). The leaves are composed of a photosynthetic leaf blade and a leaf sheath. At bulb initiation, the base of the leaf sheath swells to form the fleshy scales and the young developing leaves cease to form blades, developing instead into swollen, bladeless scales (Brewster, 1994, 2008). This provides the typical bulb structure, which is ordered according to the physiological age of the scales from inner younger to outer older scales. After maturation, one to three outer scales dry out and develop into thin, brown protective skins (Jones and Mann, 1963).

These outer skins are required to protect the bulb against disease as they provide both a physical and biochemical barrier to infection by pathogens (Currah and Proctor, 1990). In addition, the skins prevent moisture loss from the scale surface and reduce respiration rate (Apeland, 1971). Although onion skins play an important role in postharvest onion storage and shelf life, knowledge of their formation is limited (Hole et al., 2002). Onion-skin formation involves dramatic changes, including tissue drying, cell senescence and death processes, and accumulation of brown pigments in the outer scales. To date, most of the studies of onion-skin formation have focused mainly on the browning process, describing the composition of phenolic compounds in the skins. Dry skins contain high amounts of phenolic compounds, mainly quercetin and its glycosylated derivatives, relative to the inner scales of the onion bulb (Patil et al., 1995; Lee et al., 2008). These compounds are responsible for skin browning (Trammell and Peterson, 1976; Downes et al., 2009). Takahama (2004) also suggested the involvement of peroxidase-dependent oxidation of phenolic compounds in onion bulb browning.

While browning of the outer scales of onion bulb has been thoroughly studied, the possibility that cell-death processes are involved with skin formation has received far less attention. It has been reported in an early study that onion-scale cells die during the browning process (Walker and Stahmann, 1955). Bhattacharya and Pappelis (1983) previously revealed cell senescence and death in the outer epidermal cells of drying leaf bases. The exact mechanism/s underlying cell-death processes during skin formation in onion bulb remain unclear. Programmed cell death (PCD) plays a substantial role in various stages of plant development, such as differentiation, embryogenesis, xylogenesis, seed coat formation and senescence, and also in response to biotic or abiotic stress (Bozhkov et al., 2004; Lam, 2004; Della Mea et al., 2007; Turner et al., 2007; Bonneau et al., 2008). We propose that PCD is involved in skin formation in onion bulb. In the current study, the principal finding is that cell death in the outer scales is part of an organized developmental program of the onion plant which may be required for the formation of its protective skin. This is supported by our observations regarding the occurrence of DNA fragmentation, changes in cell viability, morphology and ultrastructure, and in parallel expression of PCD-related genes in the scale which has the potential to develop into a protective skin. To the best of our knowledge, this is the first report describing the occurrence of PCD-related processes during the formation of onion bulb skin.

## Materials and Methods

### Plant Material

**FIGURE 1:**
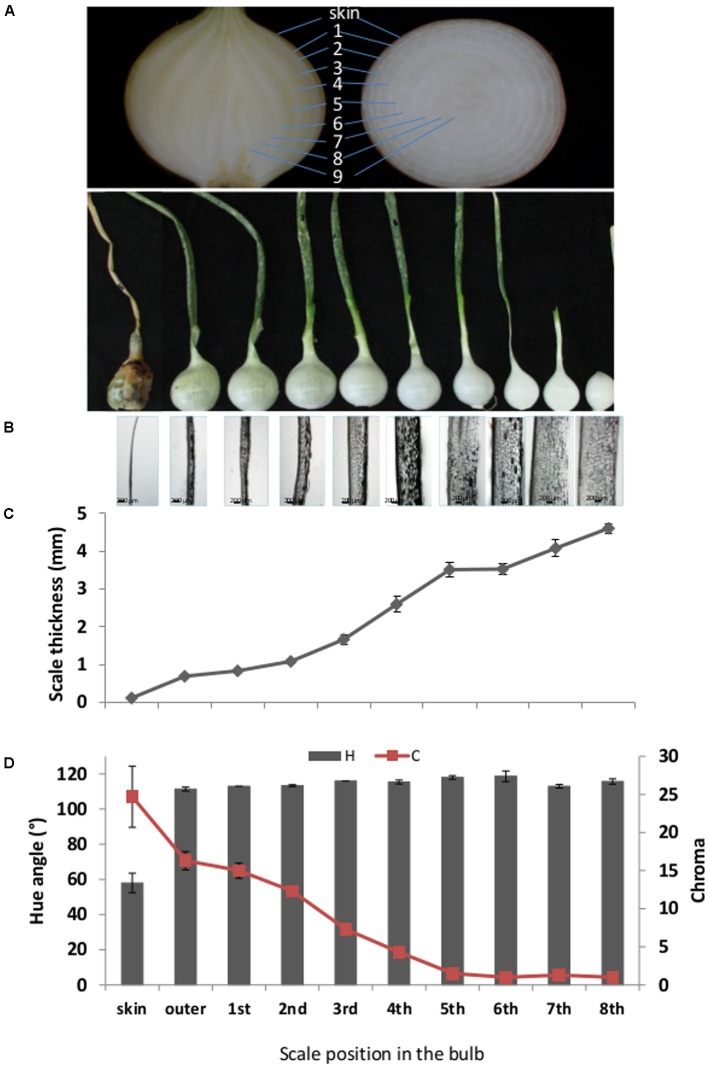
**Morphological characteristics of successive scales from exterior (left) to interior (right) of the bulb. (A)** Upper section: Representative pictures of longitude and latitude sections of onion, demonstrating the scales position. Lower section: Representative picture of separated successive scales from the outermost dry scale (left) to the innermost fleshy scale (right). Light-microscopy pictures of the corresponding successive scales. **(C)** Thickness measurements of the corresponding successive scales. **(D)** Color measurements of the corresponding successive scales represented by hue angle, H and chroma, C. Numbers 1–9 represent the scales position from the outer skin to internal part of the bulb. Error bars indicate standard error of 5 repeats, *n* = 20. Scale bar, 200 μm.

Commercial brown onion cv. Orlando was grown in sandy soil in the northwestern Negev desert in the years 2013–2015. Onions were not sprayed with maleic hydrazide before leaf drop, as per common agricultural practice, and did not undergo field curing. Onions were harvested manually at 80–100% fallen leaves (top–down) and the leaves were removed with a sharp knife, leaving a ca. 10-cm long neck above the bulb, as previously described (Eshel et al., 2014), and omitting the postharvest fast-curing process. The onions were placed in a dark storage room at 2°C and 70% relative humidity until use. Experiments were conducted with undamaged bulbs of regular shape. Bulbs freshly harvest from the field contain a single completely dry skin (“skin”) and underneath it several scales characterized as thin yellowish scales. These yellowish scales were numbered as scales 1–4 (as illustrated in **Figure 1A**), have the ability to undergo cell death and form additional dry skins. As shown in our previous publication, scales 1–4 will be developed into skin during postharvest long cold storage or in several days during Fast Curing (Eshel et al., 2014). For morphological analysis, roots were removed from the bulbs which were separated into different successive scales from exterior to interior scales of bulb (**Figure 1B**).

### TUNEL and DAPI Staining

The TUNEL reaction (TdT-mediated deoxy-uracil nick end labeling) is used to analyze DNA fragmentation by labeling the 3′-OH ends of the DNA-strand breaks. Histological analyses were performed on 10-μm-thick onion-scale sections cut by microtome, according to the method described by Teper-Bamnolker et al. (2010). For TUNEL staining, fixed tissues were rehydrated with Histoclear and decreasing concentrations of ethanol (100, 70, and 30%). Tissue permeabilization was performed with 20 μg/ml Proteinase K (Gibco BRL) in 10 mM Tris, pH 7.5, and 5 mM EDTA, pH 8.0, at 37°C for 30 min. After washing the tissue twice with phosphate-buffered saline (PBS), lysing enzyme (4 mg/ml) in 5 mM EDTA, pH 8.0, was added for 20 min with incubation at 37°C. TUNEL reaction was performed on slides using the In Situ Cell Death Detection kit with fluorescein (Roche Applied Science) according to the manufacturer’s instructions. To visualize nuclear morphology in onion cells, samples were stained with 4′-6-diamidino-2-phenylindole (DAPI; Sigma) at 1 μg/ml in PBS buffer for 10 min. Negative and positive controls were treated identically except for the omission of the enzyme solution (terminal deoxynucleotidyl transferase), or after incubation with DNAse I (Roche) for 30 min, respectively. DAPI and TUNEL-positive staining were observed with an IX81/FV500 confocal laser-scanning microscope (Olympus) equipped with a 488-nm argon ion laser and a 405-nm diode laser. DAPI was excited with the 405-nm diode laser, and the emission was filtered with BA 430–460 nm filters. TUNEL was excited with 488 nm of light, and the emission was filtered with a BA505IF filter. The transmitted light images were obtained using Nomarski differential interference contrast, and then images were subjected to Fluo View 500 software supplied with the confocal laser-scanning microscope. Percentage of TUNEL-positive cells is determine as compared to in the DAPI staining vs. cells number in the same tissue.

### Scanning Electron Microscopy (SEM)

The first four scales of the onion bulb (from the outside) were analyzed by SEM. The scales were cut into small sections (circa 5 × 5 mm), fixed in 70% ethanol overnight at room temperature and then dehydrated in ethanol solutions (90% for 1 h, 95% for 1 h, and 100% for 2 h, twice). Sections were mounted by their longest axis in vertical orientation on a slotted cryo-SEM stub and held in position using a cryo-glue (OCT) compound (Tissue Tek^®^, Sakura, Tokyo, Japan). Stubs were plunged into pre-frozen liquid nitrogen, and transferred under vacuum to a cryochamber (Alto 2100 Gatan, Abingdon, UK) with the stage temperature cooled to -180°C. Sections were fractured using a cold blade and the stage temperature was raised to 95°C to sublimate any contaminating ice and to enhance features on the fractured surface. The stage heater was turned off and once the temperature recovered to approximately 160°C, sections were coated with gold for 60 s. Prepared sections were then placed in the SEM (JSM LV6360 SEM, Jeol, Tokyo, Japan) and mounted on the stage with the temperature maintained at 160°C. The fractured surfaces were examined at ×500 magnification with an accelerating voltage of 12 kV before setting parameters for microanalysis.

### Transmission Electron Microscopy (TEM)

The 1st thin outer scale and the 5th inner fleshy scale of the onion bulb were cut into small sections (circa 3 × 3 mm) and fixed in 2.5% (w/v) glutaraldeyde in 0.1 M cacodylate buffer (pH 7.4) for 2.5 h at room temperature, and then moved to 4°C for an additional 16 h. The tissues were rinsed four times, 10 min each, in cacodylate buffer and postfixed and stained with 2% (w/v) osmium tetroxide and 1.5% (w/v) potassium ferricyanide in 0.1 M cacodylate buffer for 2 h. Tissues were then washed four times in cacodylate buffer followed by dehydration in increasing concentrations of ethanol—30, 50, 70, 80, 90, and 95%—for 10 min each step followed by 100% anhydrous ethanol three times, 20 min each, and propylene oxide twice, 10 min each. Following dehydration, the tissues were infiltrated with increasing concentrations of Agar 100 resin in propylene oxide, consisting of 25, 50, 75, and 100% resin for 16 h each step. The tissues were then embedded in fresh resin and allowed to polymerize in an oven at 60°C for 48 h. Tissues embedded in blocks were sectioned with a diamond knife on an LKB 3 microtome and ultrathin sections (80 nm) were collected onto 300-mesh, carbon/formvar-coated copper grids. The sections on the grids were sequentially stained with uranyl acetate and lead citrate for 10 min each and viewed with a Tecnai 12 TEM 100kV (Phillips, Eindhoven, the Netherlands) equipped with a MegaView II CCD camera and Analysis^®^ version 3.0 software (SoftImaging System GmbH, Munster, Germany). Representative photographs are presented.

### Fluorescein Diacetate (FDA) Staining

Tissue sections of onion scales from the first outer thin scale toward the inner fleshy scales of the bulb were hand-cut to determine cell viability. These small sections were immediately submerged in 50 μM FDA for 10 min at room temperature in the dark to maximize fluorescein formation. FDA fluorescence, indicating cell viability, was observed in a confocal laser-scanning microscope (Olympus). Excitation and emission wavelengths were 493 and 510 nm, respectively. Only cells that exhibited bright green fluorescence in the cytosol were considered viable.

### RNA Isolation, cDNA Library Construction and RNA-Seq

Three different onion scales: first outer thin scale (1st), intermediate fleshy scale (5th) and inner fleshy scale (8th), were sampled in two biological replicates and used for RNA isolation, cDNA synthesis and sequencing. Scale samples were frozen in liquid nitrogen and stored at -80°C until RNA extraction. Total RNA of each sample was extracted using the CTAB protocol (Chang et al., 1993). Samples were treated with DNase (Epicentre, Madison, WI, USA) according to the supplier’s instructions. RNA purity and integrity were verified by RNA 6000 Nano Assay on an Agilent 2100 BioAnalyzer with a minimum RNA integrity number value of 7. Library preparation and sequencing were performed at the Genome Center, Life Sciences and Engineering, Technion, Israel. Fourteen single-end RNA-Seq libraries with a length of 100 nucleotides were prepared using Illumina Hiseq2000 and Trueseq protocols.

### *De novo* Transcriptome Assembly

Raw reads were subjected to filtering and cleaning as follows: SortMeRNA tool was used to filter out rRNA (Kopylova et al., 2012) (doi: 10.1093/bioinformatics/bts611.) Then the FASTX Toolkit was used^1^ (version 0.0.13.2) for: (i) trimming read-end nucleotides with quality scores < 30 using fastq_quality_trimmer; (ii) removing reads with less than 70% base pairs with quality score ≤ 30 using fastq_quality_filter. A total of ∼270 million cleaned reads, obtained after processing and cleaning, were assembled *de novo* using Trinity software (version trinityrnaseq_r20140717 2.1.1 (Grabherr et al., 2011) with the trimmomatic option to remove adaptors (Bolger et al., 2014) and 25 mer k-mer size. The assembled sequences that shared a number of k-mers (the set of isoforms of a gene) were referred to as “contigs.” The sets of all sequences that shared at least one k-mer were referred to as components. Filtering of the likely contig artifacts and low expressed contigs was carried out as follows: (i) abundance estimates were calculated for each contig using the RSEM software (Li and Dewey, 2011); (ii) only contigs representing more than 1% of the per-component (IsoPct) expression level were retained. The resulting *de novo* assembly generated a transcriptome consisting of 45,892 contigs with N50 of 1,694, median contig length of 965 bp, and average contig length of 1,208.62 bp.

Sequencing data were deposited in the NCBI Sequence Read Archive (SRA) database as bioproject PRJNA326316 (SRX1959523, SRX1959528, SRX1959536).

### Sequence Similarity and Functional Annotation

To assess the similarity of the onion transcriptome to those of other model and closely related species, analysis of sequence similarity was performed using the BLAST (Basic Local Alignment Search Tool) algorithm with an *E*-value cut-off of 10^-5^ (Altschul et al., 1990). The BLASTX algorithm was used to search protein databases with a translated nucleotide query for comparison of the assembled contigs with sequences deposited in the databases of *Arabidopsis*^2^, *Oryza sativa*^3^ and SwissProt proteins^4^^,^^5^.

### Differential Expression and Cluster Analysis

Transcript quantification (the number of reads per gene) from the RNA-Seq data was performed using the Bowtie aligner (Langmead et al., 2009) and the RNA-Seq by Expectation-Maximization (RSEM), which handles read-mapping uncertainty with a statistical model by estimating maximum-likelihood expression levels (Li and Dewey, 2011). Differential expression analysis was performed with the edgeR software suite (Robinson et al., 2010). Transcripts that were more than fourfold differentially expressed with false discovery-corrected statistical significance of at most 0.001 and log 2 of the fold change lower than -2 or greater than 2 were considered differentially expressed (Benjamini and Hochberg, 1995). The expression patterns of the transcripts in the different samples were studied using cluster analysis of the differentially expressed transcripts in at least one pairwise sample comparison. Then following the Trinity protocol (Haas et al., 2013), expression normalization was designed using TMM (trimmed mean of *M*-values), following FPKM (fragments per feature kilobase per million reads mapped) calculations. Hierarchical clustering of the normalized gene expression [using centralized and log 2 transformation; (Haas et al., 2013)] and heatmap visualization were performed using R Bioconductor (Gentleman et al., 2004). We used the “Venny” tool (Oliveros, 2007) for Venn diagram construction.

### Gene Ontology (GO)-Enrichment Analysis

Gene ontology and Kyoto Encyclopedia of Genes and Genomes (KEGG) annotations were performed using the GSEA server^6^. GO-enrichment analysis was carried out using the Blast2GO (Conesa et al., 2005) program based on Fisher’s Exact Test (Upton, 1992) with multiple testing correction of false discovery rate (FDR) (Benjamini and Hochberg, 1995). The threshold was set to a FDR with corrected *P*-value of less than 0.05. GO analysis was performed by comparing the GO terms in the test sample to the GO terms in a background reference. GO provides a structured and controlled terminology to define gene products according to three domains: molecular function (the biochemical activity of a gene product), biological process (operations or sets of molecular events to which the gene product contributes), and cellular component (cell parts in which a gene product is active).

## Results

### Onion Scale Morphology

Peripheral onion scales are chronologically older than those located toward the center of the bulb (**Figure 1A**). To characterize the morphological differences between scales located at different positions in the bulb, we analyzed the thickness and color of sequential scales. Scale thickness increased from the outer toward the inner bulb position (**Figures 1B,C**). The thinnest tissue was the skin, and scale thickness increased gradually with a fold change of sequential scales of around 1–1.6; maximum thickness was found in the 8th inner fleshy scale (**Figures 1B,C**). Scales also differed in color. The measured hue angle (*H°*) increased dramatically from the skin toward the inner scales of the bulb. The skin had a typical brown color with *H°* value of 58, whereas the following scales had a light yellow to white color with high *H°* values of around 115 (**Figures 1A,D**). The chroma (C) which, according to McGuire (1992) represents color saturation, decreased gradually from 24.7 in the skin to close to 1 in the three innermost scales of the bulb (**Figure 1D**).

### PCD as Part of Skin Formation

**FIGURE 2:**
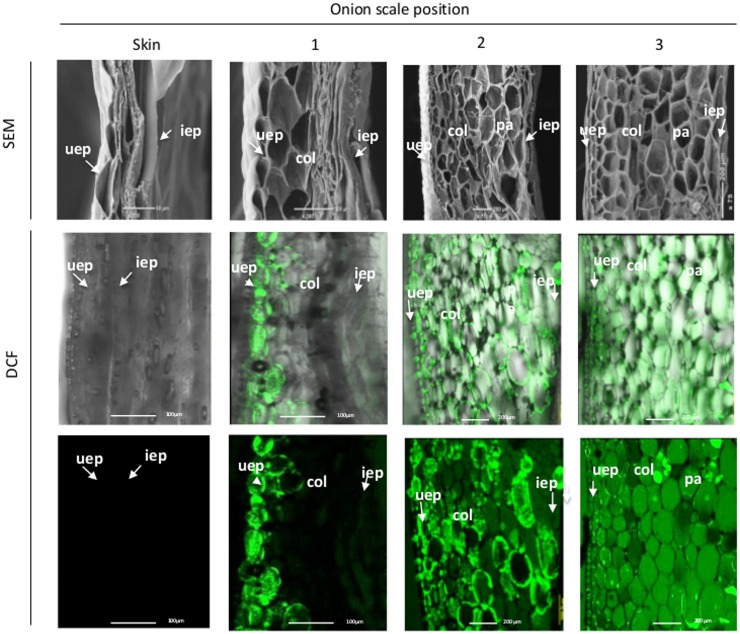
**Loss of cell viability during skin formation (right to left)**. SEM micrographs of outer onion scales (upper panels). FDA staining of the corresponding scales (lower panels). uep, upper epidermis; col, collenchyma; pa, parenchyma; iep, inner epidermis.

To characterize the differences in tissue anatomy between the different outer scales compared to the skin, SEM analysis was performed for the four chronologically ordered outer scales. The analysis revealed gradual deterioration of cells progressing from the 3rd inner scale, which included only what seemed to be intact cells, toward the skin which contained only a thin layer of collapsed cell residues (**Figure 2**). Unexpectedly, cell deterioration in the 1st outer scale initiated from the inner epidermis and progressed to the outer epidermis (**Figure 2**). Cell viability in the different scales was monitored by FDA staining. All of the cells in the 3rd inner scale were viable, whereas viability declined successively toward the skin (**Figure 2**). Within the 1st outer scale, a progressive reduction in cell viability was observed from the inner epidermis toward the parenchyma cells, while only the outer epidermis remained viable (**Figure 2**).

**FIGURE 3:**
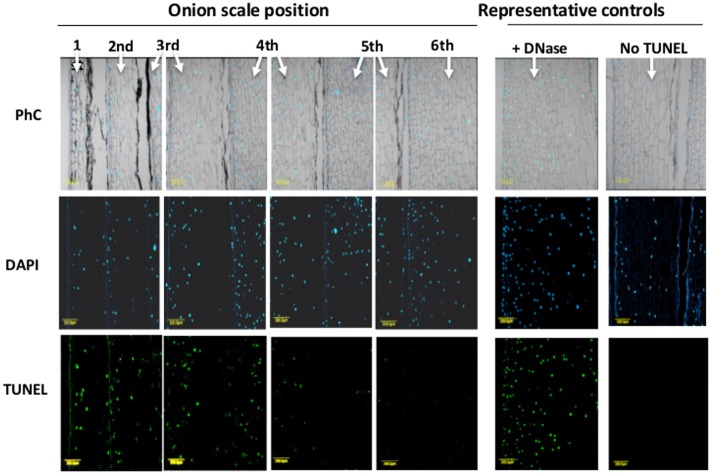
**Detection of DNA fragmentation in cross-sections of successive scales from the exterior (left) to the interior (right) of the bulb**. Histological analyses for DNA fragmentation were performed on 10-μm thick scale cross sections. The cells were counterstained *in situ* with DAPI (blue color represents nuclei) followed by TUNEL reagents (green color represents DNA fragmentation). Corresponding phase-contrast images (PhC) of scale tissues are also shown. Negative and positive controls were treated identically except for the omission of the enzyme solution (terminal deoxynucleotidyl transferase; No TUNEL) or after incubation with DNase I (Roche) for 30 min, respectively.

To detect fragmented nuclear DNA *in situ*, a TUNEL assay was applied to six scales, from outer to inner, in the same bulb. TUNEL-positive cells could be detected extensively in the 1st outer scale (around 99% of the nuclei), but their density gradually decreased from the periphery toward the center to the 6th inner fleshy scale, where no DNA fragmentation was detected (**Figure 3**). The average percentage of TUNEL-positive cells in the 1st, 2nd, 3rd, 4th, 5th, and 6th scales were 99, 93, 64, 55, and 4% respectively.

**FIGURE 4:**
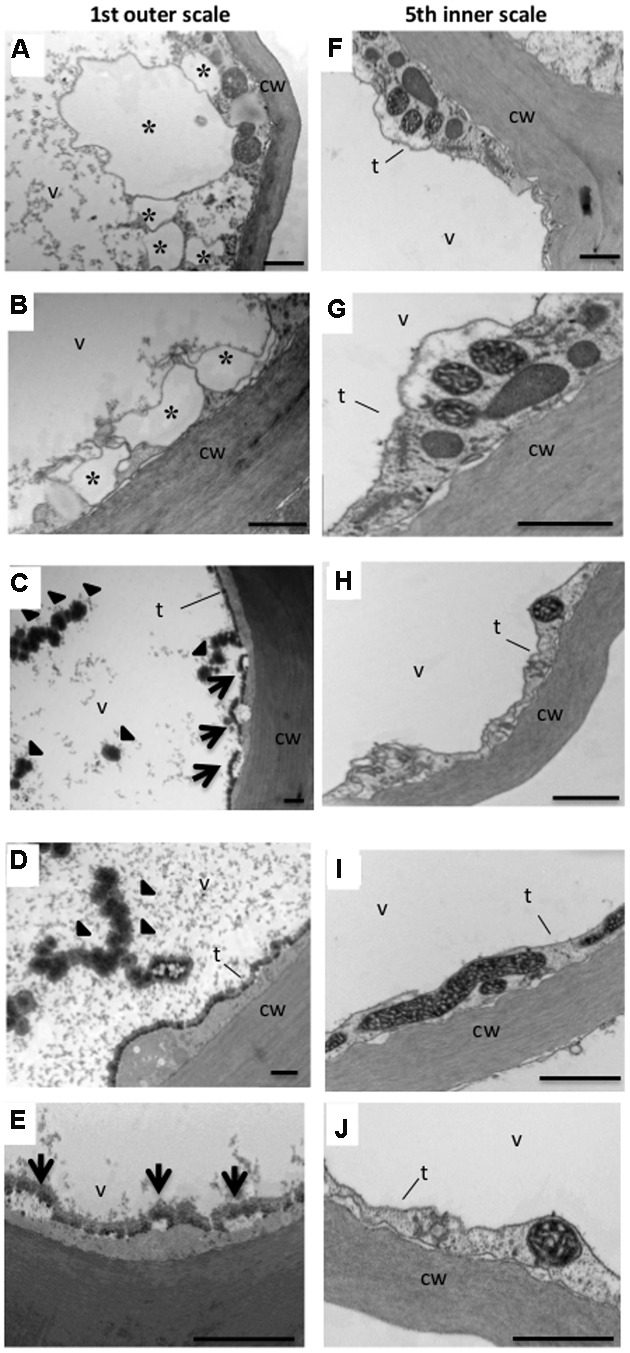
**Transmission electron micrographs of the 1st (outer) and 5th (inner) onion scales**. Note that the 1st outer scale contains a knob-like body in the vacuole and small vesicles in the cytoplasm which are not present in the 5th inner scale. (**A–E)** Parenchyma cells of the 1st outer scale. (**F–J)** Parenchyma cells of the 5th inner scale. Representative typical abnormalities in the cells are indicated by asterisks, arrows and arrowheads. Asterisks in **(A,B)** indicate small vesicles formed in the cytoplasm. Arrows in **(C,E)** indicate knob-like bodies formed on the tonoplast. Arrowheads in **(C,D)** indicate denser granular substance in the vacuole. cw, cell wall; t, tonoplast; v, vacuole. Scale bars, 1000 nm.

To examine the ultrastructural changes in the cell more closely, onion parenchyma cells of the outer thin (1st) and inner fleshy (5th) scales were analyzed by TEM. Unusual structures were found in the parenchyma cells of the outer scale which were not detected in the inner scale cells. In some parenchyma cells, there was visible formation of vesicles in the cytoplasm (**Figures 4A,B**), knob-like bodies on the surface of the tonoplast, and rupture of the tonoplast (**Figures 4C–E**). Disappearance of cytoplasm and organelles was clearly detected in the outer scale cells (**Figures 4C–E**). In addition, condensed granular substances were observed in the vacuoles in the outer scale cells (**Figures 4C,D**), but were not detected in the inner scale cells. In the latter, the organelles were clearly observable in the cytoplasm, the tonoplast seemed intact and the vacuole content was clear (**Figures 4F–J**).

### *De novo*-Assembled Transcriptome and Annotation

Six selected pools of mRNA samples, representing three scale positions: outer scale (1st), intermediate fleshy scale (5th) and inner scale (8th) of the onion bulb served for the construction of high-throughput parallel RNA-Seq libraries (**Supplementary Figure S1**). Each of the cDNA libraries yielded 16.4–20.9 million 100-bp one-end reads (**Table 1**). Quality trimming and filtration of all of the libraries resulted in ∼270 million cleaned reads that were assembled using Trinity, generating 45,891 contigs for the transcriptome catalog. The average contig length was 1208.61 bp; half of these (N50) were at least 1,694 bp long. The transcriptome catalog of onion was compared with the database of *Oryza sativa*, the most sequenced and annotated monocot species. BLASTX search against the rice database resulted in at least one significant hit for 25,703 contigs of the onion transcriptome (56%). Comparison against the TAIR (*Arabidopsis thaliana*) database resulted in significant hits for 25,398 contigs (54%). GO terms were assigned to 25,340 contigs and comparison to the KEGG database^6^ resulted in hits for 18,305 contigs. Databases of *Arabidopsis*, rice and SwissProt showed a general similarity of 54–56% with our data. InterProScan via the Blast2GO tool found 25,648 contigs with known protein motifs in the multiple databases (PROSITE, PRINTS, Pfam, ProDom, SMART, TIGRFAMs, PIR superfamily, SUPERFAMILY, Gene3D, PANTHER and HAMAP).

**Table 1:** Overview of the obtained RNA-Seq data in the different onion scales

Scale position	No. clean reads	No. mapping reads	% mapping
1st – Outer (a)	20,962,091	17,926,086	85.5
1st – Outer (b)	17,903,262	14,995,844	83.8
5th – Intermediate (a)	17,637,477	14,698,083	83.3
5th – Intermediate (b)	16,373,596	13,699,655	83.7
8th – Inner (a)	18,244,378	15,166,837	83.1
8th – Inner (b)	19,101,529	16,153,007	84.6

*^∗^a and b two replicates*.

### Differential Transcriptome of Onion Scales

**FIGURE 5:**
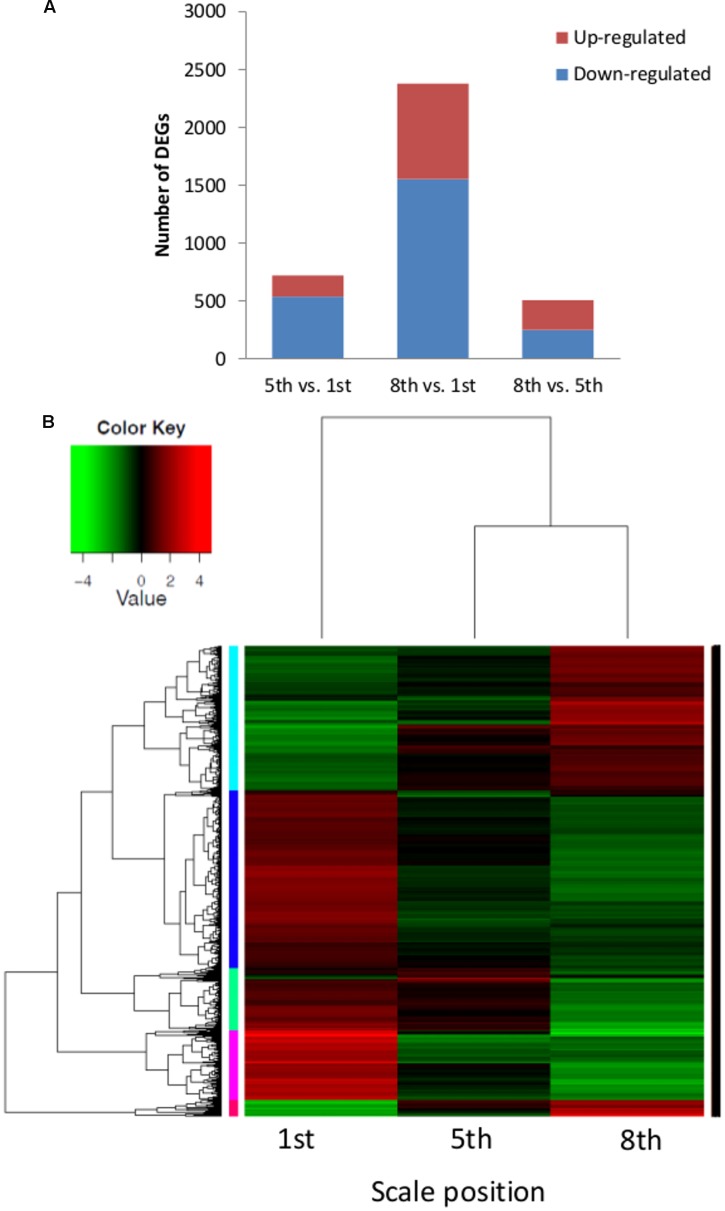
**Distribution of differentially expressed genes (DEGs) and cluster heatmap of three different onion scales (1st, 5th, and 8th). (A)** Numbers of total DEGs in pairwise comparisons of the three scales. **(B)** Heatmap and hierarchical cluster analysis of the corresponding scales. The heatmap shows the expression levels of 2,542 DEGs, their normalized expression value was centered and log 2 transformed for visualization purposes with a script taken from Trinity pipeline. Five main clusters, represented in cyan, blue, green, pink, and red, are shown.

The outer scale (1st), intermediate fleshy scale (5th) and inner scale (8th) transcriptomes were analyzed for differential gene expression. About 85% of the cleaned reads could be mapped to the *de novo* transcript catalog of onion (**Table 1**). Three pairwise combinations of the scales were compared and 2,542 significantly differentially expressed genes (DEGs) were identified. The comparison of 5th vs. 1st scales revealed 722 DEGs, 183 upregulated and 539 downregulated (**Figure 5A**). Out of 2,382 DEGs in the comparison of 8th vs. 1st scales, 827 were upregulated and 1,555 were downregulated. Finally, we identified only 509 DEGs in the comparison of 8th vs. 5th scales, 257 upregulated and 252 downregulated (**Figure 5A**). These results showed the highest proportion of DEGs between the 8th and 1st scales, an intermediate proportion between the 5th and 1st scales, and the lowest differential gene expression for the 8th vs. 5th scales (**Figure 5A**). Thus, the number of DEGs tended to increase as the distance between scales increased. Moreover, in the comparisons of either 5th or 8th scale to the 1st scale, there were more downregulated than upregulated DEGs (**Figure 5A**).

To identify shared and unique DEGs among the scales, we generated a Venn diagram based on the three comparisons. Overlap of the 5th vs. 1st scale comparison with the 8th vs. 1st scale comparison revealed 556 shared DEGs (**Supplementary Figure S2**). Thus most of the DEGs from the comparison of 5th vs. 1st scale (556 out of 722 DEGs, 70%) were included in the 8th vs. 1st scale comparison. Out of 509 DEGs in the comparison of 8th vs. 5th scale, 345 DEGs (59%) were included in the comparison of the 8th vs. 1st scale. However, only 22 DEGs overlapped between the 5th-to-1st scale comparison and the 8th-to-5th scale comparison (**Supplementary Figure S2**). These results indicated a higher difference between more distant scales. In addition, in the comparison of 8th to 1st scale, 59% of the DEGs (1,407 out of 2,832) were specific to this comparison. Only 74 DEGs were shared by all three groups.

### GO Classification of the Genes That Were Differentially Expressed in the Different Scales

**FIGURE 6:**
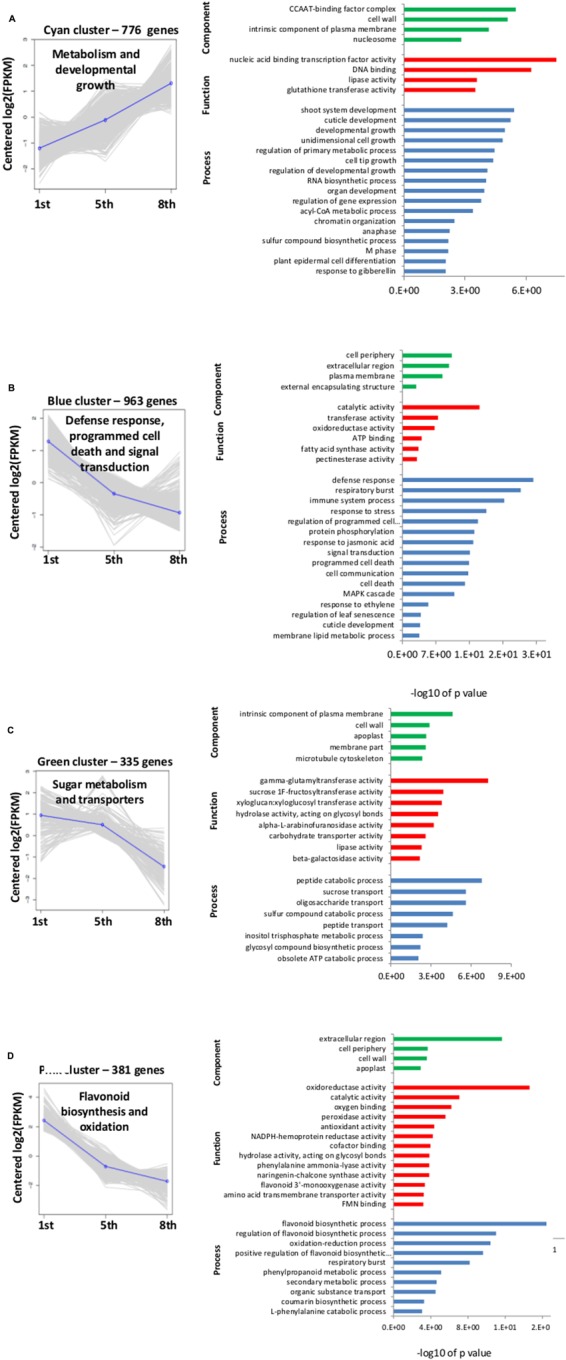
**Cluster analysis profiles and gene ontology (GO) analysis for clusters shown in **Figure 5B****. The clusters were subjected to GO-enrichment analysis, and the genes were categorized into functional groups of three main categories: biological process, cellular component and molecular function. In the left graphs, the *y*-axis indicates fold change in gene expression and the *x*-axis represents the three different onion scales (1st, 5th, and 8th). In the right graphs, the y-axis shows all the GO terms while the *x*-axis gives -log10 of *P*-values of the GO terms found in each cluster. **(A–D)** Four of the different cluster profiles obtained from hierarchical cluster analysis in **Figure 5B**.

Hierarchical cluster analysis of gene expression revealed clearly differentiated patterns of gene expression among the three investigated scales. The heatmap analysis revealed five main clusters of coexpressed genes (**Figure 5B**), each labeled in a different color according to the pattern of the difference among the three scales: cyan and red clusters describe a low expression level in the 1st scale compared to the 5th and 8th scales, whereas the blue, green and pink clusters describe high expression in the 1st scale compared to the 5th and 8th scales. We focused on four of the five clusters: cyan, blue, green, and pink, which had a common GO-term profile. The cyan cluster (776 genes) profile was the only one that included genes with higher expression in the inner 8th scale (**Figures 5B** and **6A**). GO-enrichment analysis of this cluster revealed gene-expression patterns reflecting metabolic activity, with most of the genes associated with cell growth, metabolism and developmental process (**Figure 6A**). The blue cluster (963 genes), the biggest cluster of coexpressed genes, was characterized by substantially higher expression levels in the 1st scale compared to the 5th scale, and a further moderate decrease in the inner 8th scale (**Figures 5B** and **6B**). Genes in this cluster were related to defense response, respiratory burst, and response to stress, PCD and diversity of signal transduction processes (**Figure 6B**). The green cluster (335 genes) was also characterized by decreased expression levels with a slight decrease in the 5th scale compared to the 1st, and a more significant decrease in the 8th scale (**Figures 5B** and **6C**). The most significant genes in this cluster were associated with peptide catabolic process, and sucrose and oligosaccharide transport (**Figure 6C**). These findings suggest changing carbohydrate contents in the different scales. The pink cluster (381 genes) was characterized by decreased expression levels from the 1st scale to the two inner scales (**Figures 5B** and **6D**). Most of these genes were annotated as biological processes related to pigmentation, such as flavonoid biosynthetic processes and phenylpropanoid metabolic processes (**Figure 6D**). The final, red cluster (**Figure 5B**) was relatively small, consisting of 87 genes, and lacked any common biological role. The results of this cluster are therefore not shown.

### Expression of PCD-Related Genes

**FIGURE 7:**
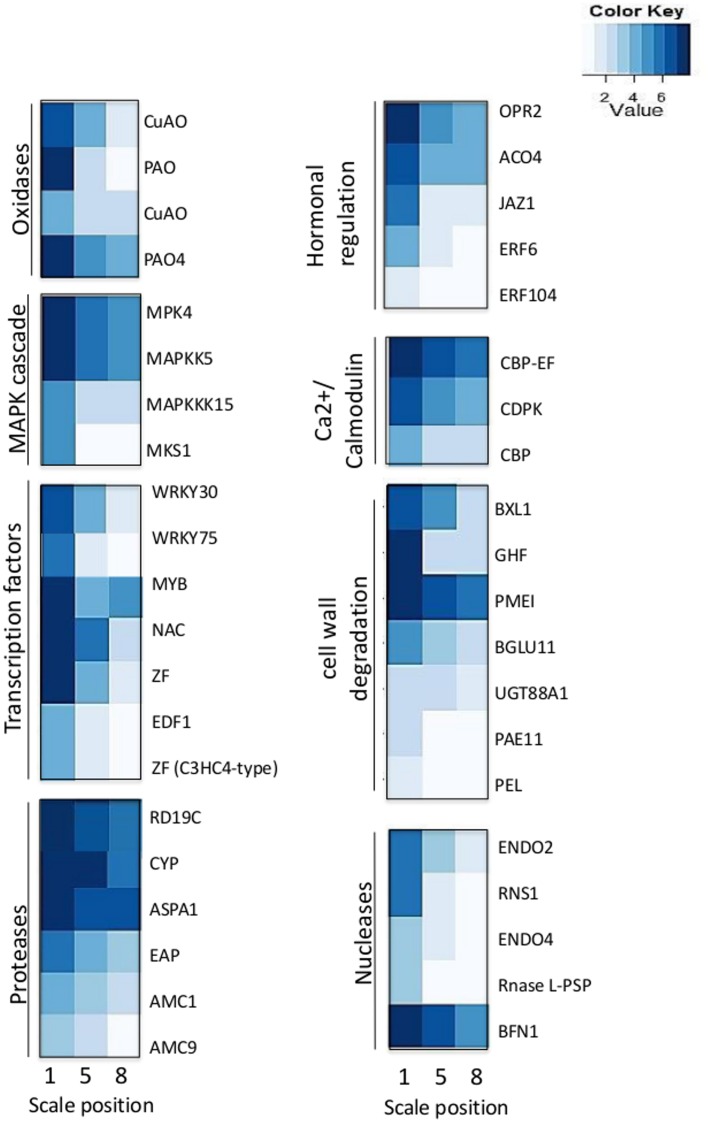
**Expression profiles of genes related to cell death in the 1st, 5th, and 8th scales**.

We analyzed the expression of PCD-related genes in the transcriptome data in the three different scales. Most of these genes were induced in the 1st outer scale and had lower expression in the 5th and 8th inner scales as shown (**Figure 7**). The PCD-related genes were subdivided into several classes; oxidases, mitogen-activated protein kinase (MAPK) cascade, Ca^2+^/Calmodulin cascade, transcription factors, proteases, hormonal regulation, signal cascades, cell wall degradation and nucleases. The oxidases genes encoding to copper amine oxidases (CuAO) and amine oxidases (POAs) were highly expressed in the 1st scale compared to the two inner scales.

Among the MAPK cascades, genes encoding to MAP kinase 4 (MPK4), MAP kinase kinase 5 (MAPKK5), MAP kinase kinase 15 (MAPKKK15) and MAP kinase substrate 1 (MKS1) were highly expressed in the 1st scale (**Figure 7**; **Table 2**). This is probably followed by expression of genes members of the Ca^2+^/calmodulin cascade such as calcium-binding EF-hand family (CBP-EF) and calmodulin-domain protein kinase (CDPK) which also were highly expressed in the 1st scale (**Figure 7**; **Table 2**). This cascades then targets various effector proteins in the cytoplasm or nucleus, which include other kinases, enzymes, or transcription factors (TFs) (Khokhlatchev et al., 1998).

**Table 2:** Description of senescence-associated PCD genes illustrated in **Figure 7**.

Gene name	Annotation	AGI number	Reference
**Oxidases**			
CuAO	Copper amine oxidase family protein	AT4G12290.1	Tavladoraki et al., 2016
PAO	FAD-linked oxidases family protein	AT5G06580.1	Tavladoraki et al., 2016
CuAO	Copper amine oxidase family protein	AT2G42490.1	Tavladoraki et al., 2016
PAO4	Polyamine oxidase 4	AT1G65840.1	Tavladoraki et al., 2016
**Hormonal regulation**			
OPR2	12-oxophytodienoate reductase 2	AT1G76690.1	Staswick, 2008; Kim et al., 2015
ACO4	1-aminocyclopropane-1-carboxylate oxidase 4	AT1G05010.1	Graham et al., 2012
JAZ1	Jasmonate-zim-domain protein 1	AT1G19180.1	Staswick, 2008; Kim et al., 2015
ERF6	Ethylene responsive element binding factor 6	AT4G17490.1	Liu et al., 2010
ERF104	Ethylene response factor 104	AT5G61600.1	Liu et al., 2010
**MAPK cascade**			
MPK4	MAP kinase 4	AT4G01370.1	Wilkins et al., 2014
MAPKK5	MAP kinase kinase 5	AT3G21220.1	
MAPKKK15	MAP kinase kinase kinase 15	AT5G55090.1	Sarwat et al., 2013
MKS1	MAP kinase substrate 1	AT3G18690.1	Damri et al., 2009
**Ca^2+^/Calmodulin**			
CBP-EF	Calcium-binding EF-hand family protein	AT1G18210.2	Jones, 2001
CDPK	Calmodulin-domain protein kinase	AT3G10660.1	Zhang et al., 2014
CBP	Calmodulin-binding family protein	AT2G26190.1	Yang and Poovaiah, 2000
**Transcription factors**			
WRKY30	WRKY DNA-binding protein 30	AT5G24110.1	Guo et al., 2004; Besseau et al., 2012
WRKY75	WRKY DNA-binding protein 75	AT5G13080.1	Guo et al., 2004; Sarwat et al., 2013
MYB	MYB domain family protein	AT3G23250.2	Guo et al., 2004; Zhu et al., 2011
NAC	NAC domain family	AT4G31550.2	Guo et al., 2004; Woo et al., 2013
ZF	Zinc-finger family	AT3G19580.2	
EDF1	AP2/ERF family	AT1G25560.1	Gutterson and Reuber, 2004
ZF (C3HC4-type)	Zinc finger (C3HC4-type RING finger) family protein	AT1G08050.1	
**Proteases**			
RD19C	Papain family cysteine protease	AT4G16190.1	Richau et al., 2012; van Wyk et al., 2014
CYP	Cysteine proteinase superfamily protein	AT5G50260.1	Jiang and Deyholos, 2010
ASPA1	Aspartic proteinase A1	AT1G11910.1	Beers et al., 2004
EAP	Eukaryotic aspartyl protease family protein	AT5G22850.1	Beers et al., 2004
AMC1	Metacaspase 1	AT1G02170.1	Coll et al., 2014
AMC9	Metacaspase 9	AT5G04200.1	Kwon and Hwang, 2013; Escamez and Tuominen, 2014
**Nucleases**			
ENDO2	Endonuclease 2	AT1G68290.1	Triques et al., 2007; Lesniewicz et al., 2013
RNS1	Ribonuclease 1	AT2G02990.1	
ENDO4	Endonuclease 4	AT4G21585.1	Triques et al., 2007; Lesniewicz et al., 2013
Rnase L-PSP	Endoribonuclease L-PSP family protein	AT3G20390.1	
BFN1	Bi-functional nuclease i	AT1G11190.1	Pérez-Amador et al., 2000; Farage-Barhom et al., 2008, 2011; Fendrych et al., 2014; Sakamoto and Takami, 2014
**Cell wall degradation**			
BXL1	β-xylosidase 1	AT5G49360.1	Lee et al., 2007
GHF	Glycosyl hydrolase superfamily protein	AT4G16260.1	Lee et al., 2007
PMEI	Pectin methylesterase inhibitor superfamily	AT4G02320.1	Lee et al., 2007
BGLU11	β-glucosidase 11	AT1G02850.2	Lee et al., 2007
UGT88A1	UDP-glucosyl transferase 88A1	AT3G16520.2	
PAE11	Pectinacetylesterase family protein 11	AT5G45280.1	
PEL	Pectate lyase family protein	AT1G67750.1	Lee et al., 2007

The targets of the MAPK signal cascade include various types of TFs. Among these, many of the genes are expressed in the 1st scale, including genes encoding WRKY DNA-binding protein 30 and 75 (WRKY30, WRKY75), MYB-domain family protein (MYB), NAC-domain family (NAC) zinc finger family such as the C3HC4-type RING, which presumably play a role in regulating specific protein levels via the ubiquitination pathway (**Figure 7**; **Table 2**). These are likely candidates for the control of later PCD processes. Proteases encoding genes were overrepresented in the 1st scale mainly due to expression of genes homologous to genes encoding papain family cysteine protease (RD19C), cysteine proteinase (CYP), Aspartic proteinase A1 (ASPA1) and metacaspases 1 and 9 (AMC1, AMC9) (**Figure 7**; **Table 2**).

Several genes homologous to PCD-associated nuclease genes, including those encoding to endonuclease 2 (ENDO2), endonuclease 4 (ENDO4) and bi-functional nuclease 1 (BFN1) were upregulated in the 1st scale. In parallel, degradation processes which were found to occur in the outer scale were associated by elevated expression of genes homologous to genes known to be involved with cell wall degradation, such as genes encoding pectinacetylesterase, involved in the degradation of plant cell wall pectin components. In addition genes homologous to genes encoding enzymes such as β-xylosidase 1 (BXL1), glucosyl hydrolase superfamily (GHF), pectin methylesterase inhibitor (PMEI), β-glucosidase 11 (BGLU11), and UDP-glucosyl transferase 88A1 (UGT88A1), were highly upregulated in the 1st scale (**Figure 7**; **Table 2**).

## Discussion

### Programmed Senescence in Outer Scales

The sequential scales of the onion bulb, which have a common origin as leaf bases, differed in various characteristics. Their thickness increased gradually from the outer to inner scales, as also revealed by light microscopy (**Figures 1B,C**). The scales also differed in color, the skin having a typical brown color, whereas the other scales were light yellow to white (**Figures 1A,D**). The brown color of the skin has been associated with peroxidase-dependent oxidation of phenolics (Takahama, 2004). Onion scales contain quercetin 4′-glucoside and quercetin 3,4′-diglucoside as major phenolics, with their concentration increasing from the inner to outer scales (Tsushida and Suzuki, 1995; Hirota et al., 1998). Outer-scale browning is a result of loss of cellular compartmentalization due to cell death, since in living cells, enzymes that catalyze glucosidation and the respective substrates may be compartmentalized (Takahama and Oniki, 2000).

Structural analysis of the outer scale demonstrated cell death within that scale that initiated from the parenchyma cells and spread gradually toward the outer epidermis (**Figure 2**). Since cell death and tissue desiccation are unique to the outer scale and start from the inner tissue, we hypothesized that these processes are active and programmed. Similarly, it has been reported that cell death in leaf senescence also initiates from mesophyll cells and then proceeds to other cell types (Lim et al., 2007). Mochizuki-Kawai et al. (2013) demonstrated that PCD begins earlier in the mesophyll cells than in the epidermal cells of tulip petals during senescence.

A typical hallmark of PCD, DNA fragmentation, was detected by TUNEL assay in the outer scales and gradually disappeared toward the inner scales (**Figure 3**). This pattern of nuclear DNA fragmentation demonstrates, for the first time, that the mechanism of cell death in the outer scales during skin formation is programmed. PCD has been detected in naturally senescing leaves from a variety of plants (Yen and Yang, 1998; Simeonova et al., 2000; Cao et al., 2003; Uzelac et al., 2008). However, to the best of our knowledge, there are no studies on PCD during skin formation, although there are studies related to the role of PCD in protective dry tissue such as seed-coat development. DNA fragmentation has been found at early developmental stages of *Arabidopsis* seed-coat formation (Nakaune et al., 2005) and during the development of seed coats of cowpea (Lima et al., 2015). Radchuk et al. (2010) identified DNA fragmentation during barley pericarp development. Differences in the frequency of TUNEL positive nuclei were also detected between the inner and outer faces of the scales. As we observed in the structural analyses, cell death proceeds gradually from the inner to the outer face of the scale. This might be explained by the loss of the nuclei in the inner face dead cells which does not allow detection by the TUNEL assay.

Our TEM analyses of ultrastructural changes in the scales supported the results of PCD in the outer scales. The irregular features of the cells in the outer scale may be involved in the process of PCD, since no such abnormalities were found in cells of the inner scale that remained viable (**Figure 4**). Similar changes, including small vesicles in the cytoplasm and granular substances in the vacuoles, have been found during developmental PCD in other species (Teper-Bamnolker et al., 2012). Gunawardena et al. (2004) reported that dying cells contain many electron-dense knob-like bodies and electron-lucent blebs on the inner surface of the tonoplast in cells undergoing PCD during remodeling of leaf shape in the lace plant. Differentiation-induced PCD occurs in various processes, for example, in xylem tracheary elements (Bollhöner et al., 2012), leaf morphogenesis (Gunawardena, 2008), floral development (Rogers, 2006), root cap formation (Fendrych et al., 2014) and anther tapetum layer formation (Van Hautegem et al., 2015). Senescence-induced PCD is the last stage of organ senescence which occurs in all tissues of an organ (Thomas, 2013). It can be suggested that the PCD in the outer scale is a developmental PCD process, as it is associated with skin formation.

### Transcriptional Regulation of Skin Formation

In this study, we found that the most overrepresented metabolic processes in the outer scale are associated with defense response, PCD processes, carbohydrate metabolism and flavonoid biosynthesis (**Figure 6**). It is well known that outer onion browning derives from loss of cellular compartmentalization due to cell death, which leads to enzymatic oxidation of phenolic compounds by peroxidase (Takahama, 2004). Upregulation of genes related to flavonoids and secondary metabolic processes may be relevant to this browning mechanism (**Figure 6**). In contrast to the outer scale, gene expression associated with increased metabolism and developmental growth processes was overrepresented in the inner scales (**Figure 6**). This difference in gene expression probably results in the inner scales having viable tissue that preserves its metabolic activity as compared to the outer scale, a distressed suicide tissue that is programmed to die and form skin.

**FIGURE 8:**
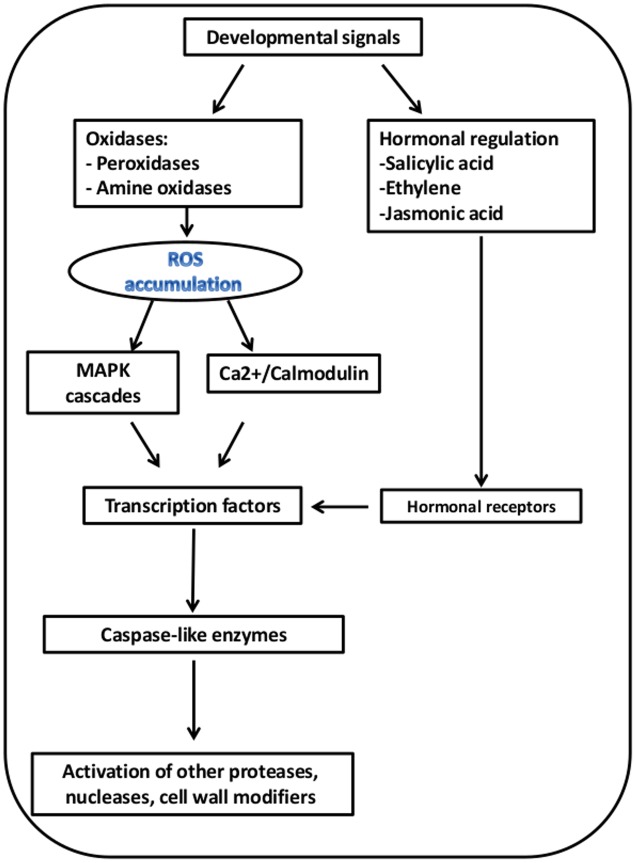
**Proposed model for PCD in the 1st outer scale**. The suggested model is based on overrepresentation of the corresponding genes in the 1st outer scales as presented in **Figure 7**.

We propose a conceptual model for PCD in the outer scale during skin formation, based on the PCD-related gene expression patterns (**Figure 7**). This hypothetical model suggests sequential processes from initiation to execution of PCD, within the outer scale (**Figure 8**). ROS-producing enzymes, including copper amine oxidases (CuAOs) and polyamine oxidases (PAOs), induce ROS accumulation (Gechev et al., 2006; Kärkönen and Kuchitsu, 2015). Apoplastic CuAOs and PAOs have been shown to play a key role as a source of ROS (such H_2_O_2_) in tissue differentiation organ development as well as PCD (Moore et al., 2003; Pourtau et al., 2006; Wingler and Roitsch, 2008; Tavladoraki et al., 2016). Sensing of ROS during senescence and PCD-related processes, activates two major signal cascades: mitogen-activated protein kinase (MAPK) and Ca^2+^/calmodulin, which leads to activation of key TFs to initiate these processes (Bieker et al., 2012; Rogers, 2012). Plant hormones have also a regulatory important role in PCD-related processes, thus genes involved in their biosynthesis and signal transduction are essential for these processes. Among these are ethylene and jasmonic acid (JA), which accelerate the processes (Altschul et al., 1990; Kim et al., 2015). We found that ethylene-response factor (ERF) genes and genes required for JA biosynthesis, such 12-oxophytodienoate reductase (OPR2) gene, and involved in controlling JA responses, such jasmonate ZIM-Domain 1 gene (JAZ1), were overrepresented in the 1st outer scale and may have crucial role in skin formation in the outer scales (**Figure 7**; **Table 2**). Ethylene is involved in developmental processes that employ PCD such as senescence (Graham et al., 2012), perforation formation in the lace plant (Dauphinee et al., 2012) and in maize endosperm development (Young et al., 1997).

In the outer senescing scale, there was overrepresentation of several genes from the MAPK cascade and the signaling cascade of calcium regulation, including genes encoding calcium and calmodulin-binding proteins. The MAPK signal cascade in plants has been shown to be involved in a wide range of cellular responses, such as biotic and abiotic stress responses, hormone responses, cell proliferation, cell death, and developmental processes (Nakagami et al., 2005). Calcium signaling may be a significant component in the regulation of senescence processes. Jones (2001) described the involvement of calcium in various types of cell death.

Various TF genes were overrepresented in the outer scale, mainly in the NAC, WRKY, MYB and zinc finger (C3HC4-type RING) families (**Figure 7**; **Table 2**). NAC and WRKY TFs have been associated with senescence in several tissues, including *Arabidopsis* leaves, petals and siliques (Miao et al., 2004; Wagstaff et al., 2009; Balazadeh et al., 2011; Besseau et al., 2012; Rogers, 2013). Several genes homologous to genes encoding different classes of proteases, including papain family cysteine protease (RD19C), cysteine proteinases (CYP), aspartic proteinase A1 (ASPA1), metacaspase 1 and 9 (AMC1, AMC9) were found to be highly expressed in the outer scale, and may have essential role as executors of PCD. In general, proteases have been shown to be involved in PCD, including cysteine proteases, serine proteases, aspartic proteases (Beers et al., 2004). Plant-specific papain-type KDEL-tailed cysteine endopeptidase (CEPs) gene has been found to be upregulated in the outer scale. This gene is similar to the Arabidopsis gene shown to be involved in PCD during vegetative development as executors for the last step in the process (Zhou et al., 2016). Aspartic proteinase PASPA3, from *Arabidopsis* was shown to be a marker of developmental PCD in root cap and seed endosperm (Fendrych et al., 2014; Fourquin et al., 2016) and other generally accepted cell death processes, e.g., the tapetum and the tracheary elements (Olvera-Carrillo et al., 2015). In plant PCD, special functions are described for metacaspases (Tsiatsiani et al., 2011). Metacaspases, which are cysteine-dependent proteinases, have been associated with various types of plant PCD, such as in controlled pathogen-induced PCD in *Arabidopsis* (Coll et al., 2010) and in embryogenesis in *Picea* (Suarez et al., 2004). In *Arabidopsis*, there are nine metacaspase genes, although only metacaspase 9 is highly expressed in senescence and PCD (Kwon and Hwang, 2013). Metacaspase 9 is also strongly upregulated in older *Arabidopsis* petals (Winter et al., 2007). At the final stages of the suggested model, PCD-associated endonuclease genes, mainly the bi-functional nuclease 1 (BFN1) gene were upregulated in the 1st outer scale. BFN1 gene, a senescence and PCD marker has been shown to be induced during both senescence and developmental PCD (Pérez-Amador et al., 2000; Fendrych et al., 2014; Sakamoto and Takami, 2014). This gene suggested to be involved in DNA degradation during senescence or PCD processes in plants (Aoyagi et al., 1998; Muramoto et al., 1999; Panavas et al., 1999). Farage-Barhom et al. (2008) demonstrated the intracellular localization of BFN1-GFP in *Arabidopsis* cells undergoing senescence and death, which initial in filamentous structures spread throughout the cytoplasm, which then clustered around the nuclei as the protoplasts senesced. This pattern of localization highlights BFN1’s function as a nucleic acid-degrading enzyme in senescence and PCD. Another recent study revealed that nuclease BFN1 is responsible for the rapid cell-autonomous corpse clearance and DNA fragmentation during *Arabidopsis* root cap cell death (Fendrych et al., 2014). Senescence-related degradation processes were reflected in the enrichment of genes for cell wall degradation in the outer scale. Several genes encode degrading-enzymes such as β-xylosidase 1 (BXL1), β-glucosidase 11 (BGLU11), pectin methylesterase inhibitor (PMEI) and UDP-glucosyl transferase 88A1 (UGT88A1), were found in the 1st outer scale, and they may have a role in scale senescence by controlling the degradation of cell wall components and releasing sugars for respiration (Lee et al., 2007). The transcriptome analysis in this work led to a putative model for senescence in the outer scale that supports the involvement of PCD. The identification of genes involved in senescence-associated PCD in the outer scale revealed a possible basis for cell-death processes in skin formation. Our results clearly demonstrate that cell death in the outer scales of onion bulbs is mediated by programmed processes accompanied by a unique set of morphological and transcriptional alterations, leading to formation of the protective dry skin.

## Author Contributions

OG, made all experiment and writing; AD-F, bioinformatic analysis; PT-B, experiments design and assistance; AD, greenhouse and field experiments; YF, TEM analysis; AL and DE, supervising, experiment design and manuscript writing.

## Conflict of Interest Statement

The authors declare that the research was conducted in the absence of any commercial or financial relationships that could be construed as a potential conflict of interest.

## Footnotes

1 http://hannonlab.cshl.edu/fastx_toolkit/index.html

2 http://www.arabidopsis.org

3 www.phytozome.org

4 http://www.uniprot.org/

5 ftp://ftp.uniprot.org/pub/databases/uniprot/current_release/knowledgebase/complete/niprot_sprot.fasta.gz

6 http://www.genome.jp/kegg/

## Supplementary Material

FIGURE S1 **Experimental design of the sequencing, assembly, annotation, construction and analyses of the scale-specific transcriptome catalogues of onion (*Allium cepa* L. cv. Orlando)**.

FIGURE S2 **Venn diagram of all the scales pairs comparisons differentially expressed genes**. Each scales pair comparison consist of up and down regulated genes.

## References

[B1] Altschul S. F. Gish W. Miller W. Myers E. W. Lipman D. J. (1990). Basic local alignment search tool. *J. Mol. Biol.* 215 403–410. 10.1016/S0022-2836(05)80360-2 2231712

[B2] Aoyagi S. Sugiyama M. Fukuda H. (1998). BEN1 and ZEN1 cDNAs encoding S1-type DNases that are associated with programmed cell death in plants. *FEBS Lett.* 429 134–138. 10.1016/S0014-5793(98)00563-8 9650576

[B3] Apeland J. (1971). Effects of scale quality on physiological processes in onion. *Acta Hortic.* 20 72–79. 10.1007/s10620-015-3651-7

[B4] Balazadeh S. Kwasniewski M. Caldana C. Mehrnia M. Zanor M. I. Xue G.-P. (2011). ORS1 an H 2 O 2-responsive NAC transcription factor, controls senescence in *Arabidopsis thaliana*. *Mol. Plant* 4 346–360. 10.1093/mp/ssq080 21303842 PMC3063519

[B5] Beers E. P. Jones A. M. Dickerman A. W. (2004). The S8 serine, C1A cysteine and A1 aspartic protease families in *Arabidopsis*. *Phytochemistry* 65 43–58. 10.1016/j.phytochem.2003.09.005 14697270

[B6] Benjamini Y. Hochberg Y. (1995). Controlling the false discovery rate: a practical and powerful approach to multiple testing. *J. R. Stat. Soc. Series B Methodol.* 57 289–300.

[B7] Besseau S. Li J. Palva E. T. (2012). WRKY54 and WRKY70 co-operate as negative regulators of leaf senescence in *Arabidopsis thaliana*. *J. Exp. Bot.* 63 2667–2679. 10.1093/jxb/err450 22268143 PMC3346227

[B8] Bhattacharya P. K. Pappelis A. J. (1983). Nucleic acid, protein, and protein-bound lysine and arginine patterns in epidermal nuclei of the mature and senescing onion bulb. *Mech. Ageing Dev.* 21 27–36. 10.1016/0047-6374(83)90013-1 6191158

[B9] Bieker S. Riester L. Stahl M. Franzaring J. Zentgraf U. (2012). Senescence-specific alteration of hydrogen peroxide levels in *Arabidopsis thaliana* and oilseed rape spring variety *Brassica napus* L. cv. MozartF. *J. Integr. Plant Biol.* 54 540–554. 10.1111/j.1744-7909.2012.01147.x 22805117

[B10] Bolger A. M. Lohse M. Usadel B. (2014). Trimmomatic: a flexible trimmer for Illumina sequence data. *Bioinformatics* 30 2114–2120. 10.1093/bioinformatics/btu170 24695404 PMC4103590

[B11] Bollhöner B. Prestele J. Tuominen H. (2012). Xylem cell death: emerging understanding of regulation and function. *J. Exp. Bot.* 63 1081–1094. 10.1093/jxb/err438 22213814

[B12] Bonneau L. Ge Y. Drury G. E. Gallois P. (2008). What happened to plant caspases? *J. Exp. Bot.* 59 491–499. 10.1093/jxb/erm352 18272922

[B13] Bozhkov P. V. Filonova L. H. Suarez M. F. (2004). Programmed cell death in plant embryogenesis. *Curr. Top. Dev. Biol.* 67 135–179. 10.1016/S0070-2153(05)67004-4 15949533

[B14] Brewster J. (1994). Onions and other vegetable Alliums. The clasification, origins, distribution and economic importance of the major vegetable crops. *Crop Prod. Sci. Hortic.* 3 1–18.

[B15] Brewster J. L. (2008). *Onions and Other Vegetable Alliums.* Wallingford: CABI.

[B16] Cao J. Jiang F. Cui K. (2003). Time-course of programmed cell death during leaf senescence in *Eucommia ulmoides*. *J. Plant Res.* 116 7–12. 12605294 10.1007/s10265-002-0063-5

[B17] Chang S. Puryear J. Cairney J. (1993). A simple and efficient method for isolating RNA from pine trees. *Plant Mol. Biol. Rep.* 11 113–116. 10.1007/BF02670468

[B18] Coll N. Smidler A. Puigvert M. Popa C. Valls M. Dangl J. (2014). The plant metacaspase AtMC1 in pathogen-triggered programmed cell death and aging: functional linkage with autophagy. *Cell Death Differ.* 21 1399–1408. 10.1038/cdd.2014.50 24786830 PMC4131171

[B19] Coll N. S. Vercammen D. Smidler A. Clover C. Van Breusegem F. Dangl J. L. (2010). *Arabidopsis* type I metacaspases control cell death. *Science* 330 1393–1397. 10.1126/science.1194980 21097903

[B20] Conesa A. Götz S. García-Gómez J. M. Terol J. Talón M. Robles M. (2005). Blast2GO: a universal tool for annotation, visualization and analysis in functional genomics research. *Bioinformatics* 21 3674–3676. 10.1093/bioinformatics/bti610 16081474

[B21] Currah L. Proctor F. J. (1990). *Onions in Tropical Regions.* Chatham: Natural Resources Institute.

[B22] Damri M. Granot G. Ben-Meir H. Avivi Y. Plaschkes I. Chalifa-Caspi V. (2009). Senescing cells share common features with dedifferentiating cells. *Rejuvenation Res.* 12 435–443. 10.1089/rej.2009.0887 20041737

[B23] Dauphinee A. Wright H. Rantong G. Gunawardena A. (2012). The involvement of ethylene in programmed cell death and climacteric-like behaviour during the remodelling of lace plant (*Aponogeton madagascariensis*) leaves. *Botany* 90 1237–1244. 10.1139/b2012-093

[B24] Della Mea M. Serafini-Fracassini D. Del Duca S. (2007). Programmed cell death: similarities and differences in animals and plants. A flower paradigm. *Amino Acids* 33 395–404. 17653819 10.1007/s00726-007-0530-3

[B25] Downes K. Chope G. A. Terry L. A. (2009). Effect of curing at different temperatures on biochemical composition of onion (*Allium cepa* L.) skin from three freshly cured and cold stored UK-grown onion cultivars. *Postharvest Biol. Technol.* 54 80–86. 10.1016/j.postharvbio.2009.05.005

[B26] Escamez S. Tuominen H. (2014). Programmes of cell death and autolysis in tracheary elements: when a suicidal cell arranges its own corpse removal. *J. Exp. Bot.* 65 1313–1321. 10.1093/jxb/eru057 24554761

[B27] Eshel D. Teper-Bamnolker P. Vinokur Y. Saad I. Zutahy Y. Rodov V. (2014). Fast curing: a method to improve postharvest quality of onions in hot climate harvest. *Postharvest Biol. Technol.* 88 34–39. 10.1016/j.postharvbio.2013.09.002

[B28] Farage-Barhom S. Burd S. Sonego L. Mett A. Belausov E. Gidoni D. (2011). Localization of the *Arabidopsis* senescence-and cell death-associated BFN1 nuclease: from the ER to fragmented nuclei. *Mol. Plant* 4 1062–1073. 10.1093/mp/ssr045 21665915

[B29] Farage-Barhom S. Burd S. Sonego L. Perl-Treves R. Lers A. (2008). Expression analysis of the BFN1 nuclease gene promoter during senescence, abscission, and programmed cell death-related processes. *J. Exp. Bot.* 59 3247–3258. 10.1093/jxb/ern176 18603613 PMC2529240

[B30] Fendrych M. Van Hautegem T. Van Durme M. Olvera-Carrillo Y. Huysmans M. Karimi M. (2014). Programmed cell death controlled by ANAC033/SOMBRERO determines root cap organ size in *Arabidopsis*. *Curr. Biol.* 24 931–940. 10.1016/j.cub.2014.03.025 24726156

[B31] Fourquin C. Beauzamy L. Chamot S. Creff A. Goodrich J. Boudaoud A. (2016). Mechanical stress mediated by both endosperm softening and embryo growth underlies endosperm elimination in *Arabidopsis* seeds. *Development* 143 3300–3305. 10.1242/dev.137224 27287798

[B32] Gechev T. S. Van Breusegem F. Stone J. M. Denev I. Laloi C. (2006). Reactive oxygen species as signals that modulate plant stress responses and programmed cell death. *Bioessays* 28 1091–1101. 10.1002/bies.20493 17041898

[B33] Gentleman R. C. Carey V. J. Bates D. M. Bolstad B. Dettling M. Dudoit S. (2004). Bioconductor: open software development for computational biology and bioinformatics. *Genome Biol.* 5:R80 10.1186/gb-2004-5-10-r80 PMC545600 15461798

[B34] Grabherr M. G. Haas B. J. Yassour M. Levin J. Z. Thompson D. A. Amit I. (2011). Full-length transcriptome assembly from RNA-Seq data without a reference genome. *Nat. Biotechnol.* 29 644–652. 10.1038/nbt.1883 21572440 PMC3571712

[B35] Graham L. E. Schippers J. H. Dijkwel P. P. Wagstaff C. (2012). “Ethylene and senescence processes,” in *Annual Plant Reviews: The Plant Hormone Ethylene* Vol. 44 ed. McManus MT (Oxford: Wiley), 305–341.

[B36] Griffiths G. Trueman L. Crowther T. Thomas B. Smith B. (2002). Onions—a global benefit to health. *Phytother. Res.* 16 603–615. 10.1002/ptr.1222 12410539

[B37] Gunawardena A. H. (2008). Programmed cell death and tissue remodelling in plants. *J. Exp. Bot.* 59 445–451. 10.1093/jxb/erm189 17947252

[B38] Gunawardena A. H. Greenwood J. S. Dengler N. G. (2004). Programmed cell death remodels lace plant leaf shape during development. *Plant Cell* 16 60–73. 10.1105/tpc.016188 14688291 PMC301395

[B39] Guo Y. Cai Z. Gan S. (2004). Transcriptome of *Arabidopsis* leaf senescence. *Plant Cell Environ.* 27 521–549. 10.1111/j.1365-3040.2003.01158.x

[B40] Gutterson N. Reuber T. L. (2004). Regulation of disease resistance pathways by AP2/ERF transcription factors. *Curr. Opin. Plant Biol.* 7 465–471. 10.1016/j.pbi.2004.04.007 15231271

[B41] Haas B. J. Papanicolaou A. Yassour M. Grabherr M. Blood P. D. Bowden J. (2013). De novo transcript sequence reconstruction from RNA-seq using the Trinity platform for reference generation and analysis. *Nat. Protoc.* 8 1494–1512. 10.1038/nprot.2013.084 23845962 PMC3875132

[B42] Hirota S. Shimoda T. Takahama U. (1998). Tissue and spatial distribution of flavonol and peroxidase in onion bulbs and stability of flavonol glucosides during boiling of the scales. *J. Agric. Food Chem.* 46 3497–3502. 10.1021/jf980294w

[B43] Hoffman C. A. (1933). Developmental morphology of *Allium cepa*. *Bot. Gaz.* 95 279–299. 10.1086/334386

[B44] Hole C. Drew R. Gray D. (2002). Skin characteristics and quality of onion cultivars given different nitrogen and irrigation treatments. *J. Hortic. Sci. Biotechnol.* 77 191–199. 10.1080/14620316.2002.11511478

[B45] Jiang Y. Deyholos M. K. (2010). Transcriptome analysis of secondary-wall-enriched seed coat tissues of canola (*Brassica napus* L.). *Plant Cell Rep.* 29 327–342. 10.1007/s00299-010-0824-x 20145934

[B46] Jones A. M. (2001). Programmed cell death in development and defense. *Plant Physiol.* 125 94–97. 10.1104/pp.125.1.94 11154305 PMC1539334

[B47] Jones H. A. Mann L. K. (1963). *Onions and Their Allies, Botany, Cultivation, and Utilization.* London: Leonarad Hill Ltd.

[B48] Kärkönen A. Kuchitsu K. (2015). Reactive oxygen species in cell wall metabolism and development in plants. *Phytochemistry* 112 22–32. 10.1016/j.phytochem.2014.09.016 25446232

[B49] Khokhlatchev A. V. Canagarajah B. Wilsbacher J. Robinson M. Atkinson M. Goldsmith E. (1998). Phosphorylation of the MAP kinase ERK2 promotes its homodimerization and nuclear translocation. *Cell* 93 605–615. 10.1016/S0092-8674(00)81189-7 9604935

[B50] Kim J. Chang C. Tucker M. (2015). To grow old: regulatory role of ethylene and jasmonic acid in senescence. *Front. Plant Sci.* 6:20 10.3389/fpls.2015.00020 PMC4310285 25688252

[B51] Kopylova E. Noé L. Touzet H. (2012). SortMeRNA: fast and accurate filtering of ribosomal RNAs in metatranscriptomic data. *Bioinformatics* 28 3211–3217. 10.1093/bioinformatics/bts611 23071270

[B52] Kwon S. I. Hwang D. J. (2013). Expression analysis of the metacaspase gene family in *Arabidopsis*. *J. Plant Biol.* 56 391–398. 10.1007/s12374-013-0290-4

[B53] Lam E. (2004). Controlled cell death, plant survival and development. *Nat. Rev. Mol. Cell Biol.* 5 305–315. 10.1038/nrm1358 15071555

[B54] Langmead B. Trapnell C. Pop M. Salzberg S. L. (2009). Ultrafast and memory-efficient alignment of short DNA sequences to the human genome. *Genome Biol.* 10:R25 10.1186/gb-2009-10-3-r25 PMC2690996 19261174

[B55] Lee E.-J. Matsumura Y. Soga K. Hoson T. Koizumi N. (2007). Glycosyl hydrolases of cell wall are induced by sugar starvation in *Arabidopsis*. *Plant Cell Physiol.* 48 405–413. 10.1093/pcp/pcm009 17234672

[B56] Lee S. U. Lee J. H. Choi S. H. Lee J. S. Ohnisi-Kameyama M. Kozukue N. (2008). Flavonoid content in fresh, home-processed, and light-exposed onions and in dehydrated commercial onion products. *J. Agric. Food Chem.* 56 8541–8548. 10.1021/jf801009p 18759442

[B57] Lesniewicz K. Karlowski W. M. Pienkowska J. R. Krzywkowski P. Poreba E. (2013). The plant S1-like nuclease family has evolved a highly diverse range of catalytic capabilities. *Plant Cell Physiol.* 54 1064–1078. 10.1093/pcp/pct061 23620482

[B58] Li B. Dewey C. N. (2011). RSEM: accurate transcript quantification from RNA-Seq data with or without a reference genome. *BMC Bioinformatics* 12:323 10.1186/1471-2105-12-323 PMC3163565 21816040

[B59] Lim P. O. Kim H. J. Gil Nam H. (2007). Leaf senescence. *Annu. Rev. Plant Biol.* 58 115–136. 10.1146/annurev.arplant.57.032905.105316 17177638

[B60] Lima N. B. Trindade F. G. Cunha M. Oliveira A. E. A. Topping J. Lindsey K. (2015). Programmed cell death during development of cowpea (*Vigna unguiculata* (L.) Walp.) seed coat. *Plant Cell Environ.* 38 718–728. 10.1111/pce.12432 25142352

[B61] Liu J. Li J. Wang H. Fu Z. Liu J. Yu Y. (2010). Identification and expression analysis of ERF transcription factor genes in petunia during flower senescence and in response to hormone treatments. *J. Exp. Bot.* 62 825–840. 10.1093/jxb/erq324 20974735 PMC3003824

[B62] Mallor C. Balcells M. Mallor F. Sales E. (2011). Genetic variation for bulb size, soluble solids content and pungency in the Spanish sweet onion variety Fuentes de Ebro. Response to selection for low pungency. *Plant Breed.* 130 55–59.

[B63] McGuire R. G. (1992). Reporting of objective color measurements. *HortScience* 27 1254–1255.

[B64] Miao Y. Laun T. Zimmermann P. Zentgraf U. (2004). Targets of the WRKY53 transcription factor and its role during leaf senescence in *Arabidopsis*. *Plant Mol. Biol.* 55 853–867. 10.1007/s11103-005-2142-1 15604721

[B65] Mochizuki-Kawai H. Shibuya K. Ichimura K. (2013). Programmed cell death begins earlier in the mesophyll cells of tulip petals than in the epidermal cells. *Postharvest Biol. Technol.* 79 9–12. 10.1016/j.postharvbio.2012.12.010

[B66] Moore B. Zhou L. Rolland F. Hall Q. Cheng W.-H. Liu Y.-X. (2003). Role of the *Arabidopsis* glucose sensor HXK1 in nutrient, light, and hormonal signaling. *Science* 300 332–336. 10.1126/science.1080585 12690200

[B67] Muramoto Y. Watanabe A. Nakamura T. Takabe T. (1999). Enhanced expression of a nuclease gene in leaves of barley plants under salt stress. *Gene* 234 315–321. 10.1016/S0378-1119(99)00193-6 10395904

[B68] Nakagami H. Pitzschke A. Hirt H. (2005). Emerging MAP kinase pathways in plant stress signalling. *Trends Plant Sci.* 10 339–346. 10.1016/j.tplants.2005.05.009 15953753

[B69] Nakaune S. Yamada K. Kondo M. Kato T. Tabata S. Nishimura M. (2005). A vacuolar processing enzyme, δVPE, is involved in seed coat formation at the early stage of seed development. *Plant Cell* 17 876–887. 10.1105/tpc.104.026872 15705955 PMC1069705

[B70] Oliveros J. C. (2007). *VENNY. An Interactive Tool for Comparing Lists with Venn Diagrams.* Available at: http://bioinfogp.cnb.csic.es/tools/venny/ [accessed November 6, 2012]

[B71] Olvera-Carrillo Y. Van Bel M. Van Hautegem T. Fendrych M. Huysmans M. Simaskova M. (2015). A conserved core of programmed cell death indicator genes discriminates developmentally and environmentally induced programmed cell death in plants. *Plant Physiol.* 169 2684–2699. 10.1104/pp.15.00769 26438786 PMC4677882

[B72] Panavas T. Pikula A. Reid P. D. Rubinstein B. Walker E. L. (1999). Identification of senescence-associated genes from daylily petals. *Plant Mol. Biol.* 40 237–248. 10.1023/A:1006146230602 10412903

[B73] Patil B. S. Pike L. M. Yoo K. S. (1995). Variation in the quercetin content in different colored onions (*Allium cepa* L.). *J. Am. Soc. Hortic. Sci.* 120 909–913.

[B74] Pérez-Amador M. A. Abler M. L. De Rocher E. J. Thompson D. M. Van Hoof A. Lebrasseur N. D. (2000). Identification of BFN1 a bifunctional nuclease induced during leaf and stem senescence in *Arabidopsis*. *Plant Physiol.* 122 169–180. 10.1104/pp.122.1.169 10631260 PMC58855

[B75] Pourtau N. Jennings R. Pelzer E. Pallas J. Wingler A. (2006). Effect of sugar-induced senescence on gene expression and implications for the regulation of senescence in *Arabidopsis*. *Planta* 224 556–568. 10.1007/s00425-006-0243-y 16514542

[B76] Radchuk V. Weier D. Radchuk R. Weschke W. Weber H. (2010). Development of maternal seed tissue in barley is mediated by regulated cell expansion and cell disintegration and coordinated with endosperm growth. *J. Exp. Bot.* 62 1217–1227. 10.1093/jxb/erq348 21059741 PMC3022404

[B77] Richau K. H. Kaschani F. Verdoes M. Pansuriya T. C. Niessen S. Stüber K. (2012). Subclassification and biochemical analysis of plant papain-like cysteine proteases displays subfamily-specific. *Plant Physiol.* 158 1583–1599. 10.1104/pp.112.194001 22371507 PMC3320171

[B78] Robinson M. D. Mccarthy D. J. Smyth G. K. (2010). edgeR: a Bioconductor package for differential expression analysis of digital gene expression data. *Bioinformatics* 26 139–140. 10.1093/bioinformatics/btp616 19910308 PMC2796818

[B79] Rogers H. J. (2006). Programmed cell death in floral organs: how and why do flowers die? *Ann. Bot.* 97 309–315. 10.1093/aob/mcj051 16394024 PMC2803638

[B80] Rogers H. J. (2012). Is there an important role for reactive oxygen species and redox regulation during floral senescence? *Plant Cell Environ.* 35 217–233. 10.1111/j.1365-3040.2011.02373.x 21635270

[B81] Rogers H. J. (2013). From models to ornamentals: how is flower senescence regulated? *Plant Mol. Biol.* 82 563–574. 10.1007/s11103-012-9968-0 22983713

[B82] Sakamoto W. Takami T. (2014). Nucleases in higher plants and their possible involvement in DNA degradation during leaf senescence. *J. Exp. Bot.* 65 3835–3843. 10.1093/jxb/eru091 24634485

[B83] Sarwat M. Naqvi A. R. Ahmad P. Ashraf M. Akram N. A. (2013). Phytohormones and microRNAs as sensors and regulators of leaf senescence: assigning macro roles to small molecules. *Biotechnol. Adv.* 31 1153–1171. 10.1016/j.biotechadv.2013.02.003 23453916

[B84] Simeonova E. Sikora A. Charzyñska M. Mostowska A. (2000). Aspects of programmed cell death during leaf senescence of mono-and dicotyledonous plants. *Protoplasma* 214 93–101. 10.1007/BF02524266

[B85] Staswick P. E. (2008). JAZing up jasmonate signaling. *Trends Plant Sci.* 13 66–71. 10.1016/j.tplants.2007.11.011 18261950

[B86] Suarez M. F. Filonova L. H. Smertenko A. Savenkov E. I. Clapham D. H. Von Arnold S. (2004). Metacaspase-dependent programmed cell death is essential for plant embryogenesis. *Curr. Biol.* 14 R339–R340. 10.1016/j.cub.2004.04.019 15120084

[B87] Takahama U. (2004). Oxidation of vacuolar and apoplastic phenolic substrates by peroxidase: physiological significance of the oxidation reactions. *Phytochem. Rev.* 3 207–219. 10.1023/B:PHYT.0000047805.08470.e3

[B88] Takahama U. Oniki T. (2000). Flavonoids and some other phenolics as substrates of peroxidase: physiological significance of the redox reactions. *J. Plant Res.* 113 301–309. 10.1007/PL00013933

[B89] Tavladoraki P. Cona A. Angelini R. (2016). Copper-containing amine oxidases and FAD-dependent polyamine oxidases are key players in plant tissue differentiation and organ development. *Front. Plant Sci.* 7:824 10.3389/fpls.2016.00824 PMC4923165 27446096

[B90] Teper-Bamnolker P. Buskila Y. Lopesco Y. Ben-Dor S. Saad I. Holdengreber V. (2012). Release of apical dominance in potato tuber is accompanied by programmed cell death in the apical bud meristem. *Plant Physiol.* 158 2053–2067. 10.1104/pp.112.194076 22362870 PMC3320206

[B91] Teper-Bamnolker P. Dudai N. Fischer R. Belausov E. Zemach H. Shoseyov O. (2010). Mint essential oil can induce or inhibit potato sprouting by differential alteration of apical meristem. *Planta* 232 179–186. 10.1007/s00425-010-1154-5 20390295

[B92] Thomas H. (2013). Senescence, ageing and death of the whole plant. *New Phytol.* 197 696–711. 10.1111/nph.12047 23176101

[B93] Trammell K. Peterson C. (1976). Quantitative differences in the flavonol content of yellow onion, *Allium cepa* L. *J. Am. Soc. Hortic. Sci.* 3 205–207.

[B94] Triques K. Sturbois B. Gallais S. Dalmais M. Chauvin S. Clepet C. (2007). Characterization of *Arabidopsis thaliana* mismatch specific endonucleases: application to mutation discovery by TILLING in pea. *Plant J.* 51 1116–1125. 10.1111/j.1365-313X.2007.03201.x 17651368

[B95] Tsiatsiani L. Van Breusegem F. Gallois P. Zavialov A. Lam E. Bozhkov P. (2011). Metacaspases. *Cell Death Differ.* 18 1279–1288. 10.1038/cdd.2011.66 21597462 PMC3172103

[B96] Tsushida T. Suzuki M. (1995). Flavonoid in fruits and vegetables, 1: isolation of flavonoid-glycosides in onion and identification by chemical synthesis of the glycosides. *J. Jpn. Soc. Food Sci. Technol.* 42 100–108. 10.3136/nskkk.42.100

[B97] Turner S. Gallois P. Brown D. (2007). Tracheary element differentiation. *Annu. Rev. Plant Biol.* 58 407–433. 10.1146/annurev.arplant.57.032905.105236 17472568

[B98] Upton G. J. (1992). Fisher’s exact test. *J. R. Stat. Soc. Ser. A Stat. Soc.* 155 395–402. 10.2307/2982890

[B99] Uzelac B. Janoševiæ D. Budimir S. (2008). In situ detection of programmed cell death in Nicotiana tabacum leaves during senescence. *Journal Microsc.* 230 1–3. 10.1111/j.1365-2818.2008.01947.x 18387032

[B100] Van Hautegem T. Waters A. J. Goodrich J. Nowack M. K. (2015). Only in dying, life: programmed cell death during plant development. *Trends Plant Sci.* 20 102–113. 10.1016/j.tplants.2014.10.003 25457111

[B101] van Wyk S. G. Du Plessis M. Cullis C. A. Kunert K. J. Vorster B. J. (2014). Cysteine protease and cystatin expression and activity during soybean nodule development and senescence. *BMC Plant Biol.* 14:294 10.1186/s12870-014-0294-3 PMC4243279 25404209

[B102] Wagstaff C. Yang T. J. Stead A. D. Buchanan-Wollaston V. Roberts J. A. (2009). A molecular and structural characterization of senescing *Arabidopsis* siliques and comparison of transcriptional profiles with senescing petals and leaves. *Plant J.* 57 690–705. 10.1111/j.1365-313X.2008.03722.x 18980641

[B103] Walker J. Stahmann M. (1955). Chemical nature of disease resistance in plants. *Annu. Rev. Plant Physiol.* 6 351–366. 10.1146/annurev.pp.06.060155.002031

[B104] Wilkins K. A. Poulter N. S. Franklin-Tong V. E. (2014). Taking one for the team: self-recognition and cell suicide in pollen. *J. Exp. Bot.* 65 1331–1342. 10.1093/jxb/ert468 24449385

[B105] Wingler A. Roitsch T. (2008). Metabolic regulation of leaf senescence: interactions of sugar signalling with biotic and abiotic stress responses. *Plant Biol.* 10 50–62. 10.1111/j.1438-8677.2008.00086.x 18721311

[B106] Winter D. Vinegar B. Nahal H. Ammar R. Wilson G. V. Provart N. J. (2007). An “Electronic Fluorescent Pictograph” browser for exploring and analyzing large-scale biological data sets. *PLoS ONE* 2:e718 10.1371/journal.pone.0000718 PMC1934936 17684564

[B107] Woo H. R. Kim H. J. Nam H. G. Lim P. O. (2013). Plant leaf senescence and death–regulation by multiple layers of control and implications for aging in general. *J. Cell Sci.* 126 4823–4833. 10.1242/jcs.109116 24144694

[B108] Yang T. Poovaiah B. (2000). An early ethylene up-regulated gene encoding a calmodulin-binding protein involved in plant senescence and death. *J. Biol. Chem.* 275 38467–38473. 10.1074/jbc.M003566200 10952977

[B109] Yen C.-H. Yang C.-H. (1998). Evidence for programmed cell death during leaf senescence in plants. *Plant Cell Physiol.* 39 922–927. 10.1093/oxfordjournals.pcp.a029455

[B110] Young T. E. Gallie D. R. Demason D. A. (1997). Ethylene-mediated programmed cell death during maize endosperm development of wild-type and shrunken2 genotypes. *Plant Physiol.* 115 737–751. 10.1104/pp.115.2.737 12223841 PMC158534

[B111] Zhang H. Liu W.-Z. Zhang Y. Deng M. Niu F. Yang B. (2014). Identification, expression and interaction analyses of calcium-dependent protein kinase (CPK) genes in canola (*Brassica napus* L.). *BMC Genomics* 15:211 10.1186/1471-2164-15-211 PMC4000008 24646378

[B112] Zhou L.-Z. Höwing T. Müller B. Hammes U. Z. Gietl C. Dresselhaus T. (2016). Expression analysis of KDEL-CysEPs programmed cell death markers during reproduction in *Arabidopsis*. *Plant Reprod.* 29 265–272. 10.1007/s00497-016-0288-4 27349421

[B113] Zhu J. Lou Y. Xu X. Yang Z. N. (2011). A genetic pathway for tapetum development and function in *Arabidopsis*. *J. Integr. Plant Biol.* 53 892–900. 10.1111/j.1744-7909.2011.01078.x 21957980

