# Supplementary figures and images for: Cellular and Molecular Changes Associated with Onion Skin Formation Suggest Involvement of Programmed Cell Death
Source: Front Plant Sci. 2017 Jan 9;7:2031. doi: 10.3389/fpls.2016.02031 (PMC5220068; doi:10.3389/fpls.2016.02031)

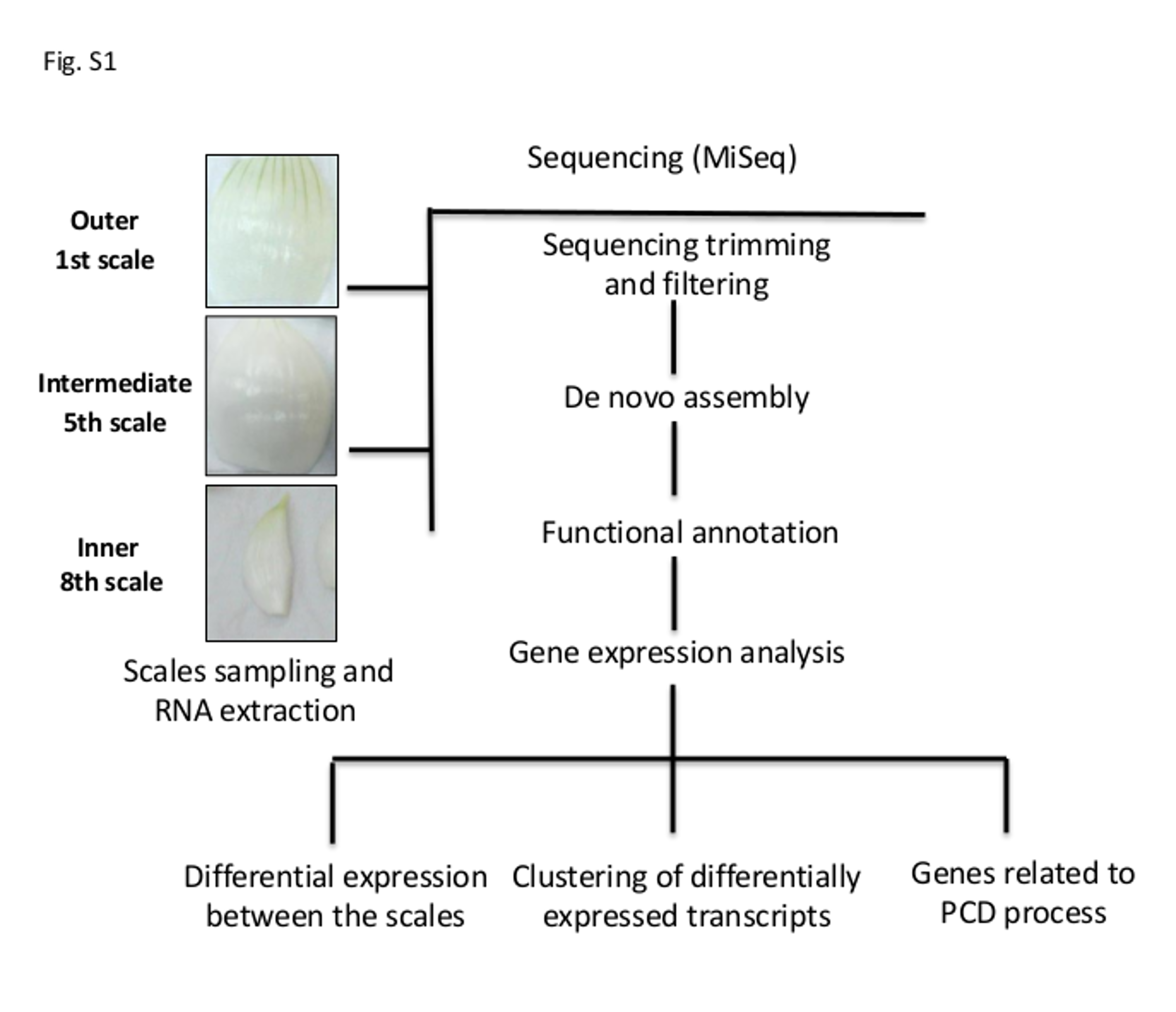

Supplement: FIGURE S1 — Experimental design of the sequencing, assembly, annotation, construction and analyses of the scale-specific transcriptome catalogues of onion (Allium cepa L. cv. Orlando). [file Image_1.tiff]

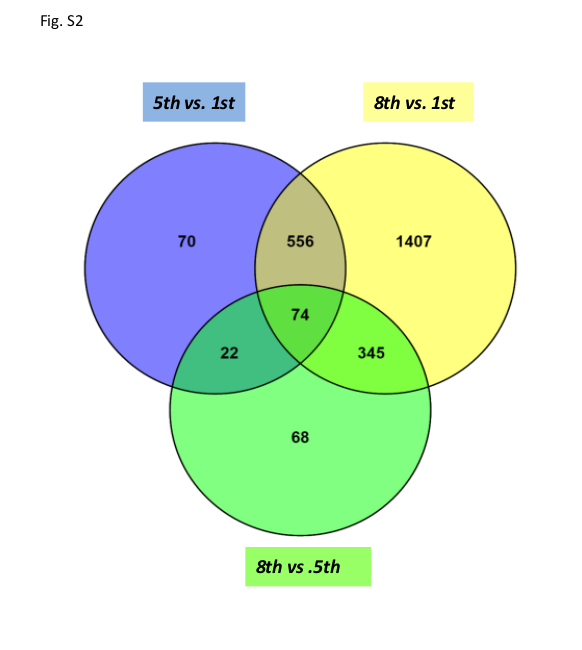

Supplement: FIGURE S2 — Venn diagram of all the scales pairs comparisons differentially expressed genes. Each scales pair comparison consist of up and down regulated genes. [file Image_2.tiff]
